# Liver Transplantation and Other Hepatically Directed Therapies Do Not Change the Biochemical Phenotype nor Halt Progression of Leukodystrophy due to Biallelic *HMBS* Variants: A Case Report

**DOI:** 10.1002/jmd2.70056

**Published:** 2025-12-09

**Authors:** Jeremy Clark, Joel Smith, Yoke Leong, Virginia Cronin, Ingrid Winship, Gayle Ross, Timothy Fazio

**Affiliations:** ^1^ Metabolic Diseases Unit Royal Melbourne Hospital Parkville Victoria Australia; ^2^ Laboratory Services Royal Children's Hospital Parkville Victoria Australia; ^3^ Department of Biochemistry Royal Melbourne Hospital Parkville Victoria Australia; ^4^ Genomic Medicine and Family Cancer Clinic Royal Melbourne Hospital Parkville Victoria Australia; ^5^ Department of Medicine The University of Melbourne Parkville Victoria Australia; ^6^ Department of Dermatology Royal Melbourne Hospital Parkville Victoria Australia

**Keywords:** acute intermittent porphyria, Heptic porphyria, *HMBS*, *HMBS* related leukodystrophy, leukodystrophy, liver transplantation

## Abstract

Leukodystrophy due to biallelic HMBS variants is a rare condition distinct from acute intermittent porphyria (AIP). It is characterised by progressive leukoencephalopathy rather than acute attacks of neurovisceral symptoms. We report the ongoing clinical progression of a patient with leukodystrophy due to homozygous variants in HMBS [c.251C>A, p.Ala84Asp] despite liver transplantation. We demonstrate that porphyrin precursor levels are unchanged following liver transplantation in the periphery and that porphyrin precursor levels are constitutively elevated in the cerebrospinal fluid and are not reduced by haem arginate therapy. Liver transplantation and hepatically directed therapies are not likely to be effective for leukodystrophy due to biallelic *HMBS* variants. Alternative treatment strategies should be developed for effective management of this disorder. One‐liner: Leukodystrophy due to biallelic *HMBS* variants is unlikely to be cured by liver transplantation or other hepatically directed therapies.

## Key Points

- Key differences between leukodystrophy due to biallelic *HMBS* variants and AIP are discussed.
- The progression of leukodystrophy due to biallelic variants in HMBS is not arrested by liver transplantation. The biochemical phenotype is not altered by liver transplantation or hepatically directed therapies.
- Neuropathology of leukodystrophy due to biallelic variants in HMBS may be due to the central production of neurotoxic porphyrin precursors.

## 1 Introduction

**FIGURE 1:**
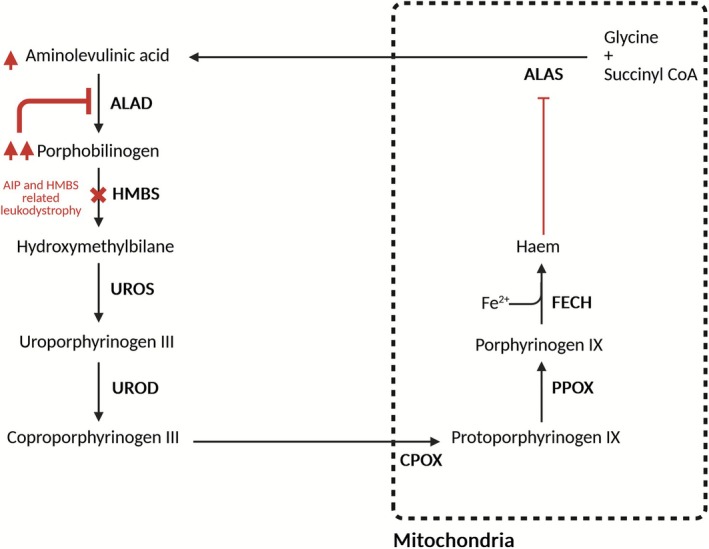
HMBS deficiency (both monoallelic and biallelic) causes porphyrin precursor accumulation. The canonical pathway for haem synthesis consists of eight enzymes. ALAS is the rate‐limiting step of hepatic haem synthesis. It is encoded by *ALAS1*. ALAS is subject to negative feedback by haem and is induced by a variety of drugs and physiological drivers. AIP and leukodystrophy related to biallelic *HMBS* variants are caused by insufficiency of enzyme function as denoted by the cross. Accumulation of porphobilinogen inhibits ALAD, leading to accumulation of aminolaevulinic acid. AIP, acute intermittent porphyria; ALAD, 5‐aminolevulinic acid dehydratase; ALAS, 5‐aminolevulinic acid synthase; CPOX, coproporphyrinogen oxidase; FECH, ferrochelatase; HMBS, HMB synthase; PPOX, protoporphyrinogen oxidase; UROD, uroporphyrinogen decarboxylase; UROS, uroporphyrinogen III synthase. Figure produced with Biorender.

Monallelic variants in *HMBS*, encoding hydroxymethylbilane synthase, are the cause of acute intermittent porphyria (AIP), an autosomal dominant condition with low penetrance. AIP is an inborn error of haem metabolism characterised by episodic attacks of neurovisceral pain caused by the accumulation of porphyrin precursors. Attacks occur after an exogenous or endogenous stimulus triggers haem synthesis, and haploinsufficiency of *HMBS* results in the build‐up of toxic, proximal precursors. 5‐aminolevulinic acid (ALA) and porphobilinogen (PBG) are thought to be the predominant toxic porphyrin precursors. In AIP, acute accumulation of these precursors exerts a toxic effect on neurological tissue through synaptic toxicity, generation of free radicals and/or impairment of mitochondrial function [1, 2]. As opposed to AIP, biallelic variants in *HMBS* cause a slowly progressive leukodystrophy. The neuropathological basis of this condition remains incompletely realised. Thirteen patients with leukodystrophy due to biallelic *HMBS* variants have been previously described. The haem synthesis pathway, and the abnormalities caused by deficiency of HMBS, are shown in Figure 1. We report the clinical course of a patient with biallelic *HMBS* variants and leukoencephalopathy, who, having undergone liver transplantation, continues to have slow, progressive neurological deterioration. This case cautions against hepatically directed therapies for this rare condition. Additionally, we report novel biochemical findings suggesting ongoing non‐hepatic production of porphyrin precursors in the periphery and elevated porphyrin precursors in the cerebrospinal fluid after transplantation.

## 2 Case Report

A 48‐year‐old female patient presented with slowly progressive ataxia, pyramidal signs and neuropathy, with onset in late childhood. She also had childhood‐onset cataracts. In 2016, she was identified as having a homozygous variant in *HMBS:* c.[251C>A];[251C>A], p.[Ala84Asp];[Ala84Asp]. The patient's diagnosis has been previously described, as has the decision to refer for liver transplantation [3].

The patient is one of five siblings born to first cousin Lebanese parents. Three siblings have a heterozygous variant in *HMBS* (c.251C>A), and one brother has biallelic variants (c.[251C>A];[251C>A], p.[Ala84Asp];[Ala84Asp]). The brother with biallelic variants shares the phenotype of leukodystrophy with prominent ataxia. His clinical course has diverged from our patient's after a pontine haemorrhage in 2016 resulted in significant superimposed neurodisability. The patient's mother and father had not had genetic sequencing prior to their deaths. No relatives of the patient have had neurovisceral symptoms of AIP. Biochemical results were not available for asymptomatic and/or deceased family members.

Our patient had normal motor development until late childhood, when the insidious development of motor and speech ataxia was noted. Assessment in 2013 noted spastic/ataxic paraparesis, length‐dependent sensorimotor peripheral neuropathy, mild dysarthria with normal cognitive function. Apparent flares of neurovisceral pain were reported, though they were fleeting, and porphyrin precursors were never measured during episodes. Diagnosis was, as above, via exome sequencing. Erythrocyte HMBS activity was measured at 18% in our patient (and in her brother with the shared genotype and phenotype) and 50% in an asymptomatic sibling carrying a monoallelic variant. Because of progressive ataxia between 2017 and 2019 (International Cooperative Ataxia Rating Scale [ICARS] score progressing from 34 to 40 [out of 100]), the patient was referred and accepted for liver transplantation, undertaken in May 2019. Transplant was uncomplicated, and a small improvement in ICARS was transiently noted. However, the patient did not experience subjective improvement and subsequently developed progressive objective deterioration in ataxia scales (ICARS 41/100 17 months post‐transplant). There was no difference in the urine PBG/Cr before or after liver transplantation. Fleeting abdominal pain, previously ascribed to AIP, recurred.

Since the response to liver transplant was less effective than expected, it was postulated that the donor may indeed also have had a variant in the *HMBS* gene. With the consent from the donors next of kin, as well as approval by the clinical ethics committee at Royal Melbourne Hospital, a single gene test of *HMBS* was undertaken. No pathogenic variants were detected.

**FIGURE 2:**
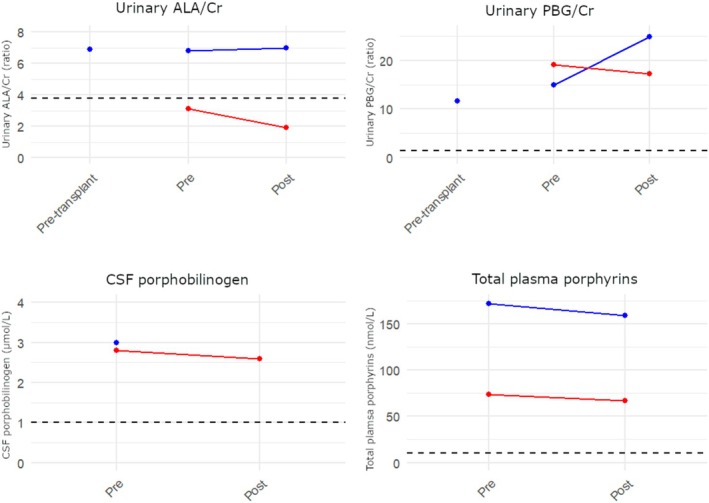
Biochemical non‐response to haem arginate in this patient with biallelic *HMBS* variants. Haem arginate was delivered intravenously for 3 days with assays measured immediately prior and the day after infusions were complete. Red line denotes the first trial of haem arginate and blue lines denote the second trial of haem arginate. The patient declined a fourth lumbar puncture (after the second infusion). Dotted black line denotes the upper limit of the 95% confidence interval in control patients. Porphyrin precursors were similar before and after liver transplantation in both peripheral and central compartments. CSF PBG levels were constitutively elevated. There was no response in either compartment to haem arginate infusion.

Our multidisciplinary porphyria team assumed the patient's care in 2020 when progressive neurological deterioration necessitated a trial of alternate therapies. A port‐a‐cath was placed to allow provision of haem arginate. The patient was given a 3‐day course of 250 mg IV haem arginate daily on two separate occasions. There was transient, subjective improvement in function during haem arginate infusions without meaningful longevity or clinical correlate. There was no difference between pre‐transplant porphyrin precursor levels and post‐transplant levels in either the peripheral or central compartment. ALA and PBG were measured in urine and CSF using the Mauzerell and Granick method [4] validated for CSF as a matrix [5]. Biochemical data is shown in Figure 2. We were not able to detect PBG in the CSF of patients unaffected by AIP. A trial of givosiran therapy was sought on a compassionate basis. Access was declined given the patient's status post liver transplant and the hepatic targeting of this therapy.

**FIGURE 3:**
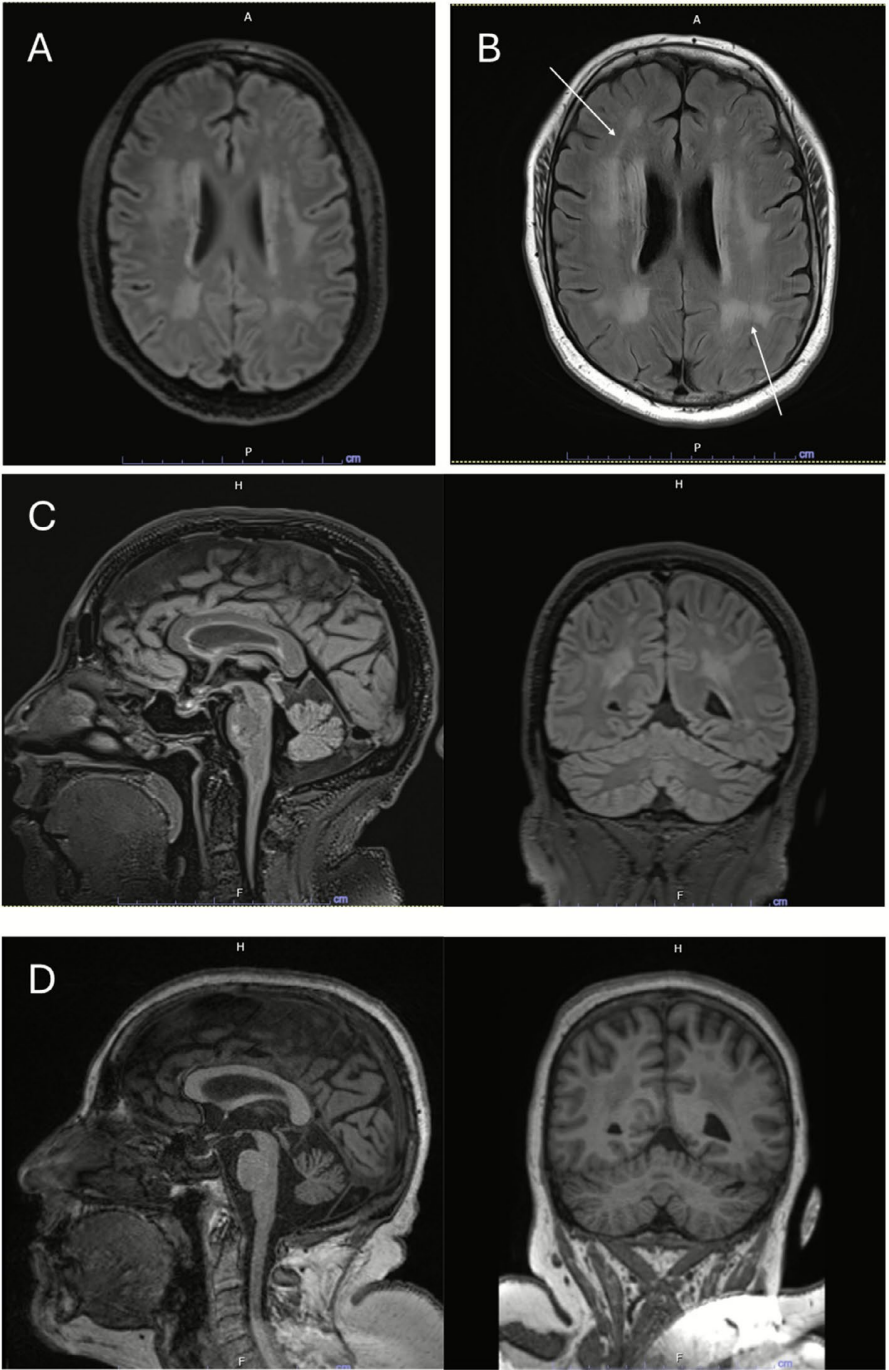
MRI demonstrating subtle progressive leukodystrophy and cerebellar atrophy. (A) and (B) are single axial slices of fluid attenuated inversion recovery (FLAIR) sequences from the initial MRI following liver transplantation (A) and the most recent follow‐up imaging in 2024 (B). Arrows demonstrate progressive confluence of periventricular T2 hyperintensities. (C) and (D) are sagittal (left) and coronal (right) slices from FLAIR sequence for the initial MRI following liver transplantation (C) and T1 weighted images from the most recent follow‐up imaging in 2024 (D). There is possible increased prominence of cisternal and subarachnoid spaces in the most recent images. Due to the transition of care there were no comparable imaging sequences to compare cerebellar size.

Assessment in 2025 noted an ICARS of 55, with ataxic/spastic paraparesis, sensory disturbance of the lower limbs and dysarthria with complete intelligibility during slow speech. Review of imaging revealed subtle progression of leukodystrophy (Figure 3).

## 3 Discussion

To the best of our knowledge, this is the only patient with biallelic *HMBS* variants to undergo liver transplantation. These are thus novel findings of persistent porphyrin precursor elevations in CSF and urine of a patient with biallelic *HMBS* variants after liver transplantation. Ongoing deterioration despite liver transplantation, and the lack of response to haem arginate therapy suggest that this is a centrally driven pathology, most likely due to central porphyrin precursor accumulation. We would caution clinicians against conflating leukodystrophy due to biallelic HMBS variants with AIP due to a monoallelic variant in *HMBS*. We would suggest that in this pathology there are potentially three key points of difference from AIP: firstly, an absence of the typical acute attacks which characterise AIP; secondly, the presence of progressive neuropathology associated with constitutively elevated porphyrin precursors in the central nervous system; and thirdly, non‐responsiveness to hepatically directed therapies.

An acute porphyria attack must have symptoms consistent with an acute attack as well as a significantly elevated PBG/Cr ratio [6]. Our patient and previously described patients with biallelic *HMBS* variants have not had acute symptoms consistent with classical acute attacks as seen in AIP, nor acute fluctuations in urinary PBG/Cr ratios or other porphyrin precursors. Animal models of biallelic *HMBS‐related* disease have also shown a similar lack of inducibility. Mice homozygous for the established pathogenic R167Q variant showed minimal biochemical response to porphyrinogenic stimuli. This is compared to the established T1/T2 mouse model (30% HMBS activity), which shows a biochemical response consistent with an acute flare of AIP. Biallelic R167Q mice have normal levels of ALAS1 mRNA transcript before and after porphyrinogenic stimuli and are non‐responsive to haem provision [7]. This is in keeping with the limited clinical experience in the 13 published patients. None of these patients have been reported to have attacks in response to classical porphyrinogenic stimuli [3, 8, 9]. Whether this lack of inducibility is related to a specific aspect of this pathophysiology or whether this is related to alternate genetic factors is not known. One should note that other genetic factors are likely to contribute to the low penetrance of monoallelic *HMBS‐related* AIP. When one compares the population frequency of known likely‐pathogenic and pathogenic variants in large population databases to the prevalence of AIP, the penetrance of pathogenic *HMBS* variants is thought to be ~1% [10]. Whereas the penetrance of pathogenic *HMBS* variants in pedigrees with AIP may be as high as 15%–30%, suggesting there are alternate genetic and environmental factors influencing the phenotype [11]. Neither of the parents nor any of the three monoallelic siblings in this family have ever described symptoms consistent with AIP. It is possible these genetic or environmental factors may contribute to the lack of acute porphyria attacks in patients with biallelic *HMBS* variants.

PBG was not detected in the CSF of control patients without AIP, whereas it was constitutively elevated in the CSF of our patient. It has been previously shown that AIP patients may have CSF PBG elevations in the order of 1.5–3 μmol/L during severe flares, while porphyrin precursor levels were very elevated in urine and blood [12, 13]. It cannot be known whether this elevation relates to hepatically derived porphyrin precursors crossing the blood‐brain barrier or increased central production during a flare. Our patient had a PBG CSF of 2.6–3 μmol/L despite having low levels of porphyrin precursors in blood. The absence of hepatic contribution to the porphyrin precursor pool in our patient suggests that the detected PBG is predominantly centrally derived. Indeed, the previously described biallelic R167Q mouse has constitutively high concentrations of PBG and ALA in brain tissue and CSF. Whereas, similar to the aforementioned AIP patients, T1/T2 mice have large increases in peripheral PBG and ALA levels in response to porphyrinogenic stimuli (56‐fold and 34‐fold in plasma) with only small increases in CSF levels (1.5‐fold) [7]. It is of note that we were not able to measure CSF ALA. ALA is an important mediator of pathology in all acute porphyrias. PBG may mediate neurotoxicity through the inhibition of δ‐aminolevulinic acid dehydratase and an increase in ALA levels [14].

The porphyrin precursor elevation in leukodystrophy due to biallelic *HMBS* variants is independent of hepatic production. In our patient, PBG and ALA levels were not different before and after orthotopic liver transplantation. PBG and ALA levels were not different before and after haem arginate administration. Lack of CSF penetration of haem has been previously established [15]. In our patient, we believe the lack of biochemical response to haem in the central compartment most likely represents non‐penetrance of haem through the blood‐brain barrier. This mirrors the lack of response to haem of the biallelic R167Q mouse. It is important to recall that the mechanism of haem therapy is to reduce the transcription of ALAS1 [16], which is not increased in the mouse model of this condition and may not be significantly upregulated to the degree previously observed in acute attacks of AIP if this condition is a fixed bottleneck rather than an inducible condition. A lack of clinical improvement and ongoing porphyrin precursor elevations following liver transplantation in a patient with biallelic variants in *HMBS* has been previously alluded to, though not reported in detail [17]. There may be similarities between this condition and δ‐aminolevulinic acid dehydratase (ALAD) deficiency porphyria. This ultrarare condition is caused by biallelic variants in *ALAD*. Marked elevation of porphyrins in the peripheral compartment and ongoing clinical deterioration despite liver transplantation has been previously reported in a patient with ALAD deficiency pophyria [18]. Givosiran therapy was not trialled in this case. It must be noted that this siRNA against ALAS1 is conjugated to an *N*‐acetylgalactosamine moiety and is thus hepatically targeted [19]. Given the lack of response to haem therapy and liver transplantation, we would not expect biochemical or clinical response to givosiran.

Haem deficiency cannot be excluded as a possible driver of neuropathology. Though, in the biallelic R167Q mouse, brain haem content was ~80% that of the wild‐type mice and CNS histopathology was normal [7]. Perhaps more likely is the possibility that the very low level of HMBS activity forms a new pathological bottleneck in the haem synthesis pathway leading to accumulation of PBG, and therefore ALA, in vulnerable neuronal tissue.

## 4 Conclusion

These findings show that orthotopic liver transplant and hepatically directed therapies are not likely to be effective for leukodystrophy due to biallelic *HMBS* variants. These findings suggest that biallelic variants in *HMBS* confer a different phenotypic profile to AIP, with a most likely different pathological basis. This condition will require a centrally directed therapy to affect the apparently poor outcomes.

## Author Contributions

**Jeremy Clark:** conception and design, collecting data, analysis/interpretation of data, writing manuscript, reviewing manuscript. **Joel Smith:** collecting data, analysis/interpretation of data, drafting of article. **Yoke Leong:** collecting data, analysis/interpretation of data, drafting of article. **Virginia Cronin:** collecting data, analysis/interpretation of data, drafting of article. **Ingrid Winship:** analysis/interpretation of data, drafting of article. **Gayle Ross:** analysis/interpretation of data, drafting of article. **Timothy Fazio:** conception and design, analysis/interpretation of data, drafting of article.

## Funding

The authors have nothing to report.

## Consent

The patient signed consent for publication approved by The Royal Melbourne Hospital. The patient consented to the publication of this case report.

## Conflicts of Interest

The authors declare no conflicts of interest.

## Data Availability

Data supporting the results reported in this article is not publicly available and is stored within the hospital and pathology electronic medical records. Further information regarding these results could be made available on request.
